# Risk of lymph node metastasis and feasibility of endoscopic submucosal dissection in undifferentiated-type early gastric cancer

**DOI:** 10.1186/s12876-023-02771-x

**Published:** 2023-05-23

**Authors:** Pengyue Zhang, Tingting Xu, Hui Feng, Zhen Zhu, Jingjing Wang, Yalei Wang

**Affiliations:** 1 Department of Gastroenterology, The First Affiliated Hospital of Anhui Medical University, Hefei, 230022 China; 2 Department of Gastroenterology, The Second Affiliated Hospital of Anhui Medical University, Hefei, 230022 China; 3 Department of Pathology, The First Affiliated Hospital of Anhui Medical University, Hefei, 230022 China

**Keywords:** Undifferentiated, Gastric cancer, Lymph node metastasis, Endoscopic submucosal dissection, Retrospective analysis

## Abstract

**Background:**

Whether endoscopic submucosal dissection (ESD) applies to undifferentiated-type early gastric cancer (UEGC) remains controversial. We aimed to analyze the risk factors for lymph node metastasis (LNM) in UEGC and evaluate the feasibility of ESD.

**Methods:**

This study included 346 patients with UEGC who underwent curative gastrectomy between January 2014 and December 2021. Univariate and multivariate analyses of the correlation between clinicopathological features and LNM were conducted, and the risk factors for exceeding the expanded ESD indications were evaluated.

**Results:**

The overall LNM rate in UEGC was 19.94%. Among the preoperatively assessable factors, submucosal invasion (odds ratio [OR] = 4.77, 95% confidence interval [CI]: 2.14–10.66) and > 2 cm(OR = 2.49, 95% CI: 1.20–5.15) were independent risk factors for LNM, while postoperative independent risk factors were > 2 cm (OR = 3.35, 95% CI: 1.02–5.40) and lymphovascular invasion(OR = 13.21, 95% CI: 5.18–33.70). Patients who met the expanded indications had a low LNM risk (4.1%). Additionally, tumors located in the cardia (*P* = 0.03), non-elevated type (*P* < 0.01) were independent risk factors for exceeding the expanded indications in UEGC.

**Conclusions:**

ESD may be applicable for UEGC meeting the expanded indications, and preoperative evaluation should be cautious when the lesion is non-elevated type or located in the cardia.

**Trial registration:**

Chinese Clinical Trial Registry (12/05/2022 ChiCTR2200059841).

**Supplementary Information:**

The online version contains supplementary material available at 10.1186/s12876-023-02771-x.

## Background

Early gastric cancer (EGC) is defined as tumor tissue confined to the mucosa or submucosa, regardless of lymph node metastasis (LNM) [1]. The presence or absence of LNM affects not only the selection of the treatment modality but also the patient’s prognosis [2]. Endoscopic resection, represented by endoscopic submucosal dissection (ESD), is unanimously recommended by international guidelines because of its low surgical trauma, fewer complications, and complete preservation of tissue and organ function [3]. ESD has gradually become an effective alternative for EGC without LNM. Therefore, the preoperative evaluation of LNM is particularly important.

Evidence is now available that the risk of LNM in EGC can be predicted based on lesion size, depth, histological type, ulceration, and lymphovascular invasion (LVI) [4]. However, the possibility of endoscopic treatment for undifferentiated-type EGC (UEGC) is controversial because of its relatively high risk of LNM compared with differentiated EGC [5]. The previous guidelines listed non-ulcerative undifferentiated intramucosal carcinoma and tumor size of ≤ 2 cm as the expanded indication of ESD [6], and the recent Japanese guidelines included it in the absolute indication [7]; however, ESD for UEGC is still not widespread in China because of the lack of confidence in curative resection by both endoscopists and patients. This study aimed to analyze the correlation between the clinicopathological features and LNM in UEGC and to explore the feasibility of ESD in a Chinese population with UEGC.

## Methods

### Patients

This study retrospectively included 346 patients with UEGC who underwent curative gastrectomy with intraoperative lymph node dissection at The First Affiliated Hospital of Anhui Medical University between January 2014 and December 2021. All included patients underwent partial gastrectomy with intraoperative lymph node dissection and were initially diagnosed and reviewed by two pathologists as having UEGC. The exclusion criteria were as follows: (1) history of radiochemotherapy; (2) remnant gastric cancer or recurrent cancer of the remnant stomach; (3) other malignant tumors of the stomach, such as gastrointestinal stromal tumors, lymphomas, and neuroendocrine tumors; (4) serious hematologic diseases and other diseases that may affect the study results. This study followed the ethical principles of medical research involving human subjects in the Declaration of Helsinki and was approved by the ethics committee of our hospital(Quick-PJ2022-05-37). We have also registered with the Chinese Clinical Trial Registry (12/05/2022 ChiCTR2200059841).

### Data collection

Using the hospital electronic medical record system, clinicopathological data were collected as comprehensively as possible, about the selection of candidate variables, we took into account the actual clinical demands and also referred to other similar studies, including sex, age, body mass index (BMI), smoking history, drinking history, tumor size and location, based on the Japanese classification of gastric carcinoma [8]. The preoperative hematological indexes mainly included the latest preoperative hematological examination of neutrophils, lymphocytes, monocytes, platelets, hemoglobin (Hb), albumin, and tumor markers (carcinoembryonic antigen, carbohydrate antigen CA19-9, and CA125), peripheral blood neutrophil-to-lymphocyte ratio (NLR), platelet-to-lymphocyte ratio (PLR), and monocyte-to-lymphocyte ratio (MLR).

The main outcome variables were measured and classified as follows: the tumor size was calculated based on the longest diameter of the tumor on histology. According to the guidelines of the Japanese Gastric Cancer Association [8], tumor location was classified as follows: cardia, corpus (including fundus), incisura angularis, and antrum. The depth of invasion was divided into two categories: mucosal (M) and submucosal (SM). According to World Health Organization (WHO) criteria, the histological types included in the study were poorly differentiated adenocarcinoma (*por*), mucinous adenocarcinoma (*muc*), signet ring cell carcinoma (*sig*), and their mixed type. According to the Paris classification [9], the macroscopic type was divided into three groups: elevated type (type 0-I, 0-IIa, or a combination of these two types), flat (0-IIb), and depressed type (0-IIc, 0-III, or a combination of these types). The depth of the ulcer was up to or beyond the mucous membrane, and an active ulcer or scar was considered an ulcer on endoscopy. All surgically resected specimens were at 5-mm intervals, and the lymph nodes were sliced into two segments. When the tumor cells occurred in the tubular space arranged by endothelial cells or in the wall of blood vessels, LVI was considered. LNM was analyzed and judged by pathologists based on the results of hematoxylin-eosin staining.

### Statistical analysis

All statistical analyses were carried out using SPSS 26.0 software (IBM Inc, New York). Continuous data in accordance with the normal distribution are expressed as the mean ± standard deviation, those that do not conform to the normal distribution are expressed by the median and quartile spacing, and categorical data are expressed as the number of cases and frequency (%). In the univariate analysis, continuous data were compared using the t-test or Mann–Whitney U test, and categorical data were compared using the chi-square test or Fisher’s exact test. Variables with P < 0.05 in the univariate analysis were included in the binary logistic regression for multivariate analysis and were considered statistically significant at P < 0.05 (two-sided).

## Results

### Clinicopathological characteristics

The study included 346 patients who underwent total or subtotal gastrectomy, all of whom were pathologically diagnosed with UEGC. The study population comprised 187 males and 159 females, with a mean age of 56.3 ± 10.8 years, with individuals aged ≥ 60 years accounting for 139 cases and individuals aged ≤ 59 years accounting for 207 cases. There were 190 cases of lesions measuring ≤ 2 cm and 156 cases with lesions measuring > 2 cm, including 153 cases of por (44.22%), 42 cases of muc (12.14%), 19 cases of sig (5.49%), 85 cases of por mixed with signet ring cells (24.57%), and 47 cases of por with mucinous components (13.58%).

### Risk factors for LNM in UEGC

LNM occurred in 69 of 346 patients (19.94%), and there were no statistically significant differences in age, sex, smoking and alcohol history, NLR, MLR, tumor markers, location, and histological type in the LNM (+) group compared with the LNM (-) group (P > 0.05). There were significant differences between the two groups in terms of the BMI, PLR, Hb, and macroscopic type. Their size was larger, and the proportions of ulceration, SM infiltration, and LVI were also significantly higher (P < 0.05) (Table 1).

**Table 1:** Incidence of lymph node metastasis and clinicopathological characteristics in undifferentiated-type early gastric cancer

	LNM(-)	LNM(+)	X^2^	P value
**Gender**			2.88	0.089
Female	121	38		
Male	156	31		
**Age(years)**			2.46	0.116
< 60	160	47		
≥ 60	117	22		
**Smoking**			0.767	0.381
Absence	222	52		
Presence	55	17		
**Drinking**			0.042	0.838
Absence	230	58		
Presence	47	11		
BMI(kg/m^2^)	22.44 ± 3.68	21.01 ± 4.47		0.031
NLR	1.89(1.41–2.53)	1.92(1.56–3.15)		0.156
PLR	123.19(91.61-162.08)	143.54(113.56-183.87)		0.014
MLR	0.19(0.15–0.26)	0.20(0.157–0.29)		0.192
Hb(g/L)	132(119–144)	123(106-138.5)		0.005
CEA(ng/ml)	1.86(1.2–3.1)	1.66(1.12–2.16)		0.163
CA125(U/ml)	9.43(7.05–13.7)	9.25(6.16–15.97)		0.673
CA199(U/ml)	8.98(5.31–13.55)	8.41(6.05–13.76)		0.127
**Alb**(g/L)	42.91 ± 4.31	42.20 ± 4.21		0.431
**Size(cm)**			10.34	0.001
≤ 2	164	26		
> 2	113	43		
**Ulcer**			11.32	0.001
Absence	147	21		
Presence	130	48		
**Location**			4.25	0.23
cardia	21	1		
corpus	67	16		
Incisura angularis	87	21		
antrum	102	31		
**Macroscopic type**			6.39	0.04
elevated	18	3		
flat	63	7		
depressed	196	59		
**Pathological type**			4.01	0.405
sig	17	2		
por	117	36		
muc	37	5		
por + muc	38	9		
por + sig	68	17		
**Depth**			18.95	< 0.001
M	169	22		
SM	108	47		
**LVI**			83.29	< 0.001
Absence	259	34		
Presence	18	35		

BMI: body mass index;NLR: neutrophil-to-lymphocyte ratio;PLR: platelet-to-lymphocyte ratio;MLR: monocyte-to-lymphocyte ratio;Hb: hemoglobin;Alb:albumin;por:poorly differentiated adenocarcinoma,muc:mucinous adenocarcinoma, sig: signet ring cell carcinoma;M: mucosal;SM:submucosal; LVI: lymphovascular invasion;LNM: lymph node metastasis;

### Multivariate analysis of risk factors for LNM in UEGC

**Fig. 1:**
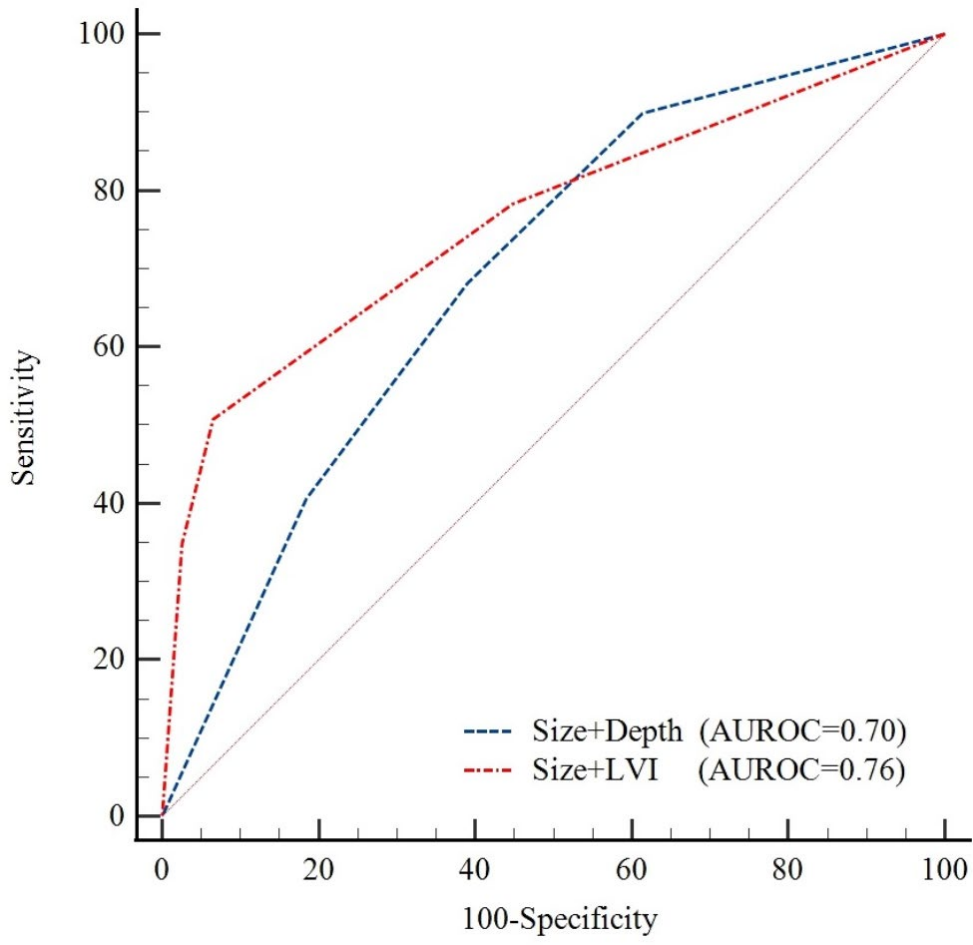
ROC curve of the risk prediction for lymph node metastasis of undifferentiated-type early gastric cancer LVI: lymphovascular invasion; AUROC:Area under the receiver operating characteristic curve

With LNM as the outcome variable, as shown in Table 2, in which factors that could be assessed by laboratory tests and endoscopy before surgery were included in the preoperative group (except LVI), and postoperative assessable factors including LVI, the results of the preoperative analysis showed that lesions measuring > 2 cm (odds ratio [OR] = 2.49, 95% confidence interval [CI]: 1.20–5.15) and SM infiltration (OR = 4.77, 95% CI: 2.14–10.66) were independent risk factors for LNM (P < 0.05); among the postoperative factors, size (OR = 3.35, 95% CI: 1.02–5.40) and LVI (OR = 13.21, 95% CI: 5.18–36.70) were independent risk factors, especially LVI, presenting the highest OR. Postoperative risk factors(size + LVI) were better than preoperative factors(size + depth) in predicting LNM based on multivariate analysis(AUROC 0.76 vs. 0.70), but the difference was not significant(Z = 1.89, *P* = 0.59)(Fig. 1).

**Table 2:** Multivariate logistic regression analysis for lymph node metastasis in undifferentiated-type early gastric cancer

Factor	Preoperative		Postoperative	
	OR(95%CI)	*P*	OR(95%CI)	*P*
BMI	0.94(0.85–1.04)	0.229	0.94(0.84–1.06)	0.329
PLR	1.00(0.99–1.01)	0.240	1.00(0.99–1.01)	0.537
Hb(g/L)	0.99(0.98–1.01)	0.178	1.00(0.98–1.01)	0.808
**Macroscopic type**				
flat	1(Ref.)		1(Ref.)	
elevated	0.33(0.03–3.70)	0.370	0.15(0.01–2.06)	0.154
depressed	0.77(0.20–2.94)	0.705	1.14(0.26–4.93)	0.861
**Size(>2 cm)**	2.49(1.20–5.15)	0.014	3.35(1.02–5.40)	0.044
**UL(+)**	1.87(0.67–5.21)	0.233	1.46(0.48–4.42)	0.506
**Depth(SM)**	4.77(2.14–10.66)	< 0.001	2.46(0.99–6.10)	0.052
**LVI(+)**			13.21(5.18–33.70)	< 0.001

BMI: body mass index;PLR: platelet-to-lymphocyte ratio;Hb: hemoglobin;UL: ulcer;SM:submucosal; LVI: lymphovascular invasion; OR: odds ratio;CI:Confidence intervals

### Subgroup analysis of LNM in UEGC

LNM in UEGC was stratified according to three risk factors, including size, depth, and ulceration, in which size and depth were independent risk factors in the multivariate analysis, and ulceration was included in the guidelines, as detailed in Table 3. The results showed that for lesions measuring ≤ 1 cm, regardless of ulceration, no postoperative LNM in the included participants (0/39), undifferentiated intramucosal cancer without ulceration, and lesions measuring ≤ 2 cm, which met the expanded indications for ESD, had a comparatively low rate of LNM (4.10%). When the lesion was ≤ 3 cm, the risk of LNM was higher than before, even for ulcer-negative intramucosal carcinoma (5.05%). When the lesion infiltrated the SM, the risk of LNM was as high as 20% (1/5), even for ulcer-negative intramucosal carcinoma measuring ≤ 1 cm, which was not suitable for endoscopic treatment.

**Table 3:** Rate of lymph node metastasis according to three risk factors [% (n)]

Size (cm)	M				SM			
	UL-	95%CI(%)	UL+	95%CI(%)	UL-	95%CI(%)	UL+	95%CI(%)
≤ 1	0(0/24)	0	0(0/15)	0	20.0(1/5)	35.5–75.5	10.0(1/10)	-12.6-32.6
> 1,≤2	6.1(3/49)	-0.8-13.1	15.4(4/26)	0.5–30.2	15.0(3/20)	2.1–32.1	34.1(14/41)	19-0.49.3
> 2,≤3	7.7(2/26)	-3.3-18.7	27.3(6/22)	7.1–47.5	7.7(1/13)	-9.1-24.5	30.6(11/36)	14.7–46.4
>3	31.2(5/16)	5.7–58.8	15.4(2/13)	-7.3-38.1	40.0(6/15)	11.9–68.1	66.7(10/15)	39.6–93.7

M: mucosal;SM:submucosal;UL:ulcer

### Risk of exceeding the ESD expanded indications in UEGC

**Fig. 2:**
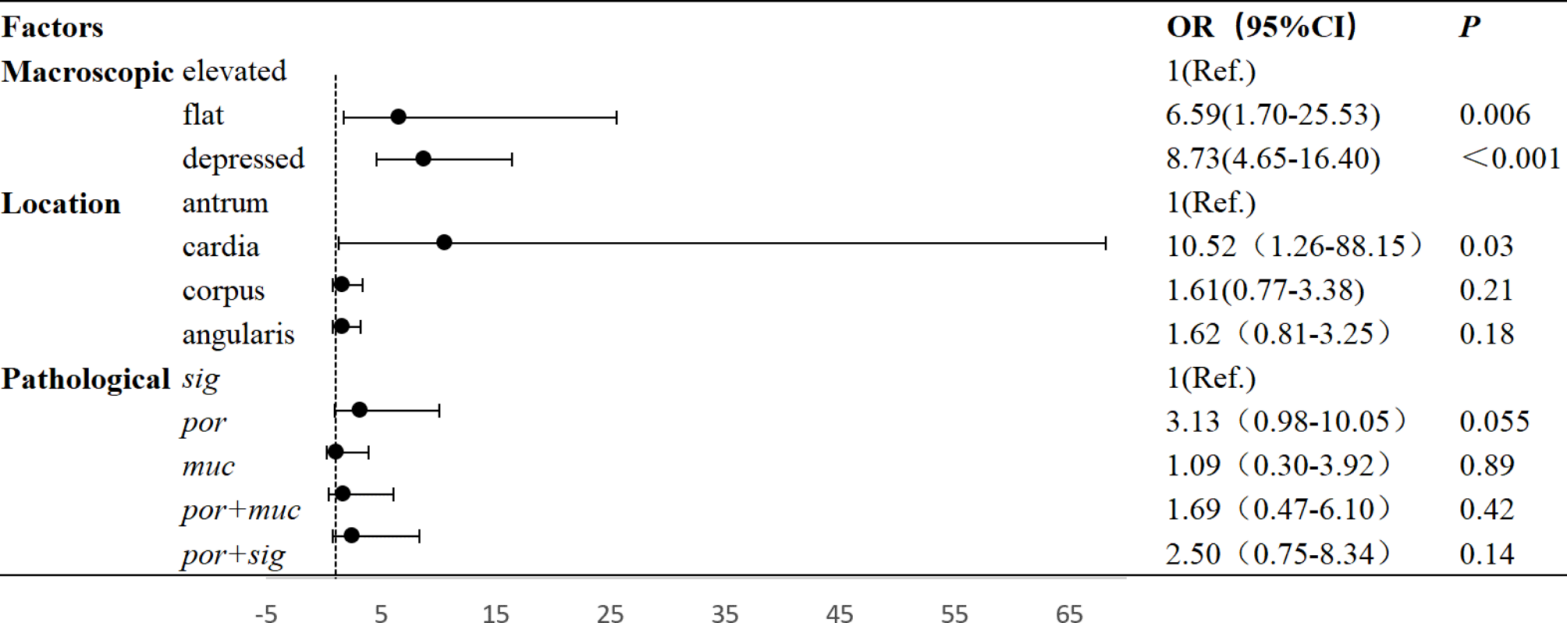
Multivariate logistic regression analysis of undifferentiated-type early gastric cancer according to endoscopic submucosal dissection indications. por:poorly differentiated adenocarcinoma,muc:mucinous adenocarcinoma, sig: signet ring cell carcinoma;LNM: lymph node metastasis; OR: odds ratio;CI:Confidence intervals

According to the guidelines, among the 346 cases included in this study, a total of 73 cases met the expanded indication, and 273 cases exceeded the indications. The univariate analysis showed that the macroscopic type, location, and pathological type were associated with exceeding the expanded indications (P < 0.05), Their multivariate analysis are presented in Fig. 2, the multivariate regression analysis showed that depressed or flat lesions(non-elevated type) and tumor located in the cardia were independent risk factors for exceeding the expanded indications (P < 0.05) (Supplementary 1).

### Characteristics of patients with LNM in expanded indications

Further analysis showed that three cases (4.10%) in the ESD indications group still had LNM, including two cases invading the M but not the SM; the clinicopathological features are listed in Table 4. All lesions were flat, two were cases of por mixed with mucinous components, and the other was a case of pure muc. Notably, the largest diameter in all three lesions was 2.0 cm, which may be a specific feature to differentiate from lymph node-negative patients.

**Table 4:** Characteristics of undifferentiated-type early gastric cancer with endoscopic submucosal dissection extended indication but with lymph node metastasis

	Gender	Age(years)	Size(cm)	Location	Depth	Form	Pathological type	LVI
1	male	82	2.0*1.5	Incisura angularis	mm	flat	muc	positivity
2	female	46	2.0*1.5	antrum	lp	flat	por + muc	negative
3	male	61	2.0*1.0	corpus	mm	flat	por + muc	negative

por:poorly differentiated adenocarcinoma,muc:mucinous adenocarcinoma, mm:muscularis mucosa;lp:lamina propria; LVI: lymphovascular invasion

## Discussion

As one of the top five malignant tumors worldwide in terms of morbidity and mortality, gastric cancer has adversely affected human health [10]. Owing to the widespread eradication of Helicobacter pylori, the incidence of intestinal gastric cancer has shown a decreasing trend, and UEGC, represented by por and sig, has gained attention [11]. As UEGC has a higher risk of LNM and a relatively poor prognosis [12], preoperative assessment of the risk of LNM should be particularly cautious. Our study showed that lesions measuring > 2 cm and SM infiltration were preoperatively independent risk factors for LNM, patients who met the expanded indications had a relatively low risk of LNM, and endoscopic resection was still possible for intramucosal carcinomas of < 1 cm, even with ulceration.

In agreement with previous studies [13, 14], our study showed that UEGC occurred mostly in the lower and middle part of the stomach, with a flat and depressed form, and the LNM rate was 19.94%, slightly higher than the 11.3% reported by Lee IS [15]. In agreement with other studies [16], LVI is the most prominent independent risk factor of LNM. As for preoperatively assessable factors, an increased risk of LNM was observed when the size of the lesion was > 2 cm. We cannot ignore lesions that are near the cut-off value in size,in our study, three patients who were eligible for ESD based on the expanded indications but presented with LNM had lesions measuring 2.0 cm, due to postoperative specimen shrinkage, the actual size may exceed 2 cm.

In addition, the LNM rates of UEGC in the M and SM were 11.5% and 30.3%, respectively, which is comparable to those reported in previous studies (11.2% and 33.0%, respectively) [17]. There is also a difference in the risk of LNM between the SM1 and SM2 or deeper, but since this study mainly included surgical specimens retrospectively, it is regrettable that no further submucosa subdivision could be performed. Our study also demonstrated that the macroscopic type and ulceration were associated with LNM; however, they were not independent risk factors. No relevance was found between the history of smoking, alcohol consumption, chronic diseases, tumor markers, and LNM, and this is consistent with the findings of Minghan Ren [18]. Similar to another study [19], PLR showed statistical differences in the univariate analysis but not in the multivariate analysis. In addition, BMI and Hb in the LNM group were lower than those of the control group. A study [20] on biopsy specimens of EGC with undifferentiated components also confirmed that BMI is a protective factor for LNM, which may be related to energy consumption along with tumor expansion.

Regarding the pathological type, a previous study [17] suggested that the rate of LNM of *sig* was similar to that of differentiated EGC and significantly lower than that of UEGC. Our study found no LNM in *sig* smaller than 2 cm regardless of the ulceration and pure *por* smaller than 2 cm without ulceration. This suggests that the risk of LNM in pure *sig* and *por* may be low, which has been confirmed in several studies [15, 21, 22]. This difference does not preclude an association with promoter CpG island hypermethylation [23]; however, due to the small sample size, no statistical difference was observed in the present study. In additional to the risk of LNM in *sig*, tumors larger than 2 cm have an increased risk of incomplete resection due to underestimation of their size, and endoscopic resection should be performed with caution [24]. Moreover, patients that meet the expanded indications for ESD but present with LNM comprise a mucinous component. A Korean study [25] also concluded that the risk of LNM is higher in *muc* than in other UEGC because of extracellular mucins which can act as a medium to promote tumor cell infiltration [26].

For intramucosal carcinoma measuring < 1 cm, regardless of whether there is an ulcer or not, no LNM was observed in our data, and endoscopic treatment was relatively suitable. We confirmed that the rate of LNM meeting the expanded indications for ESD was 4.1%, higher than the Korean analogous study (1.56%) [27]. this may be related to the difference in the pathological diagnostic system after surgery between China and Korea, and most Korean pathologists refer to Japanese standards to diagnose cancer based on nuclear and architectural heterogeneity, which is more aggressive compared with the WHO standards; thus, have a lower risk of LNM.

Although pathological criteria were not fully harmonized, patients meeting the expanded indications for ESD have a lower risk than the serious postoperative complications [28, 29] in our study. Long-term survival of patients with ESD and surgical operation also did not show clear differences (propensity score matching, *P* = 0.33) [30]. Takizawa’s multicenter study [31] further confirms the safety and efficacy of ESD in patients with UEGC based on the expanded indications. In our center, we performed ESD in 13 patients with UEGC (until Dec. 2021), two patients were lost to follow-up. Additional surgery were performed in 7 cases, one of them had positive LNM(1/7). The other 4 cases were followed up until now, only 1 case had distant LNM. There were no LNM(+) in patients with expanded indications for ESD (Supplementary 2). With the gradual convergence of pathological diagnostic criteria in Japan, endoscopic resection of UEGC that meets the expanded indications has become possible in China.

Owing to the unique biological behavior of UEGC, it is still challenging to determine its size and depth using electronic staining endoscopy and endoscopic ultrasonography [32–34]. This study suggests that tumors located in the cardia (*P* = 0.03) and depressed-type or flat-type tumors (*P* < 0.001) were independent risk factors for exceeding the expanded indications. Ohara [35] also showed that depressed lesions are independently associated with LVI and SM infiltration. Owing to the greater constriction at the cardia, even with adequate gas injection, the lesions are difficult to observe with non-extended signs under endoscopy, and the depth is easily underestimated. When the lesion has the above characteristics, caution should be exercised when assessing its indications for ESD preoperatively.

At present, there are few large sample studies on the risk of LNM in UEGC in China, and our study, being a single-center retrospective study, does not exclude the problems of selection bias. Inadequate postoperative specimen retrieval and shallow depth judgment are other limitations of this study. Besides, this is a retrospective study in which patients did not undergo preoperative endoscopic ultrasonography, which has been shown to be valuable for the assessment before ESD [36]. Also limited by the retrospective study design, the depth of infiltration and ulceration involved were derived from postoperative pathological findings, which is slightly different from the preoperative endoscopic evaluation.

## Conclusion

We believe that ESD can be considered for undifferentiated intramucosal carcinoma measuring < 2 cm without ulceration, and the risk of exceeding the expanded indication is high when the macroscopic type is non-elevated type or the lesion is located in the cardia. ESD should be evaluated carefully, and the treatment strategy should be further confirmed by high-quality survival and prognostic studies.

## Electronic supplementary material

Below is the link to the electronic supplementary material.

Supplementary Material 1

Supplementary Material 2

## Data Availability

The datasets generated or analyzed during this study are available from the corresponding author on reasonable request.
