# Supplementary figures and images for: Risk of lymph node metastasis and feasibility of endoscopic submucosal dissection in undifferentiated-type early gastric cancer
Source: BMC Gastroenterol. 2023 May 23;23:175. doi: 10.1186/s12876-023-02771-x (PMC10204219; doi:10.1186/s12876-023-02771-x)

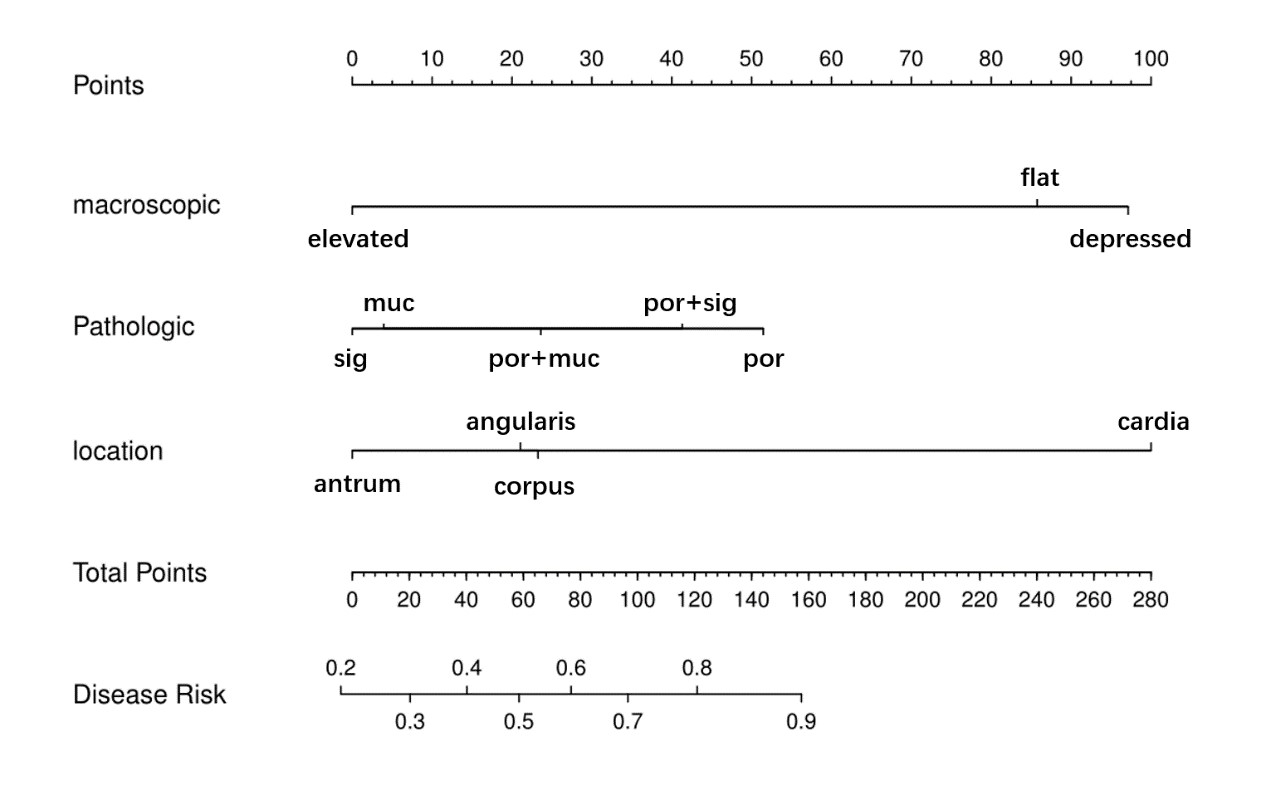

Supplement: Supplementary file 1 — Supplementary Material 1 [file 12876_2023_2771_MOESM1_ESM.jpg]

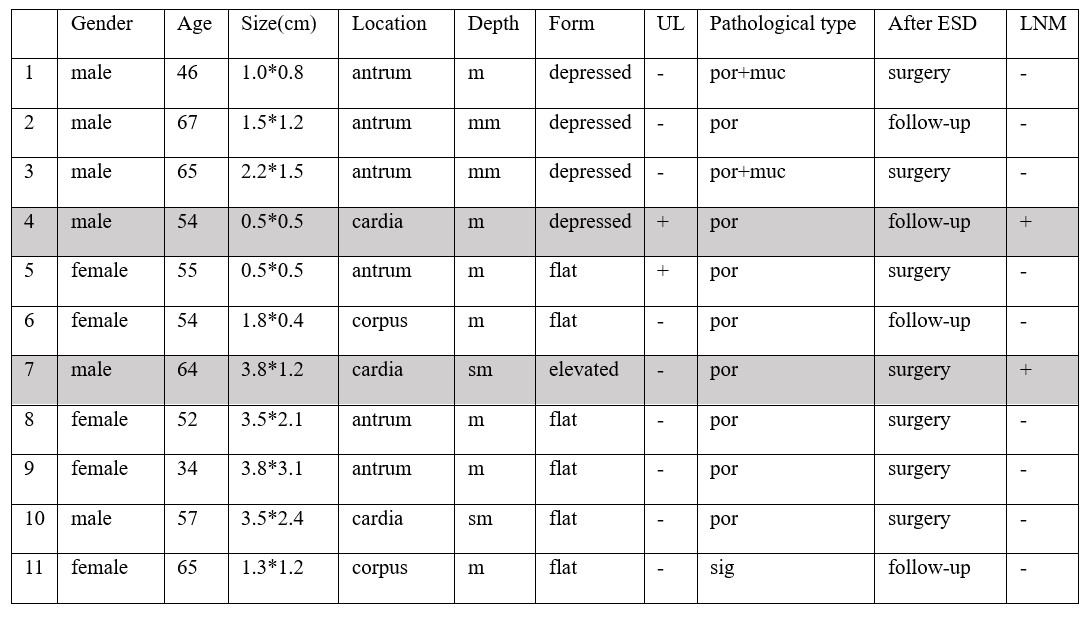

Supplement: Supplementary file 2 — Supplementary Material 2 [file 12876_2023_2771_MOESM2_ESM.jpg]
